# Swordtail Fry Attend to Chemical and Visual Cues in Detecting Predators and Conspecifics

**DOI:** 10.1371/journal.pone.0000118

**Published:** 2006-12-27

**Authors:** Seth W. Coleman, Gil G. Rosenthal

**Affiliations:** 1 Department of Biology, Texas A & M University, College Station, Texas, United States of America; 2 Centro de Investigación Científica de las Huastecas “Aguazarca”, Calnali, Hidalgo, Mexico; University of Exeter, Cornwall Campus, United Kingdom

## Abstract

Predation pressure and energy requirements present particularly salient opposing selective pressures on young fish. Thus, fry are expected to possess sophisticated means of detecting predators and resources. Here we tested the hypotheses that fry of the swordtail fish *Xiphophorus birchmanni* use chemical and visual cues in detection of predators and conspecifics. To test these hypotheses we presented young (<7 day-old) fry with combinations of visual and chemical stimuli from adult conspecifics and predators. We found that exposure to predator odors resulted in shoal tightening similar to that observed when fry were presented with visual cues alone. In trials with conspecific stimuli, fry were particularly attracted to adult conspecifics when presented simultaneous visual and chemical stimuli compared to the visual stimulus alone. These results show that fry attend to the odors of adult conspecifics, whose presence in a particular area may signal the location of resources as well as an absence of predators. This is one of the first studies to show that such young fish use chemical and visual cues in predator detection and in interactions with conspecifics. Previous research in *X. birchmanni* has shown that anthropogenic alteration of the chemical environment disrupts intraspecific chemical communication among adults; we suggest that because fry use the same chemosensory pathways to detect predators and conspecifics, alteration of the chemical environment may critically disrupt predator and resource detection.

## Introduction

Predation pressure is a primary force driving morphological, physiological, and behavioral evolution in many fishes [1]–[6]. As a result, individuals in prey species frequently employ multiple sensory systems – visual, chemical, auditory, tactile – in the detection and avoidance of predators. Vulnerability to predators may be particularly high for young fish due to their small size and lack of experience with predators. In two-spotted gobies (*Gobiusculus flavescens*), for instance, whether juveniles respond to predator chemical stimuli depends on prior visual experience with the predator [7]; [see also ref. 8]. Moreover, young fish may experience constant and conflicting pressure imposed by the high metabolic demands of growth and development countered by foraging-associated predation risk. Thus, young fish are expected to attend closely to cues associated with predation risk while foraging [e.g. ref. 9], and to modify their behavior appropriately when these cues are encountered.

In addition to attending to cues associated with threat, young fish should also attend to cues associated with resource availability and the absence of threat. We suggest that one way they may do this is to attend to the odors of adult conspecifics, whose presence in a particular area may signal the location of resources as well as an absence of predators. Here we investigate whether young fry of the swordtail fish *Xiphophorus birchmanni* attend to chemical and visual cues in the detection of predators and conspecifics.

*X. birchmanni* is a small livebearing species that inhabits shallow, rocky streams of the Río Pánuco Basin in Mexico [10], [11]. While these sexually dimorphic fish have been of much recent interest to evolutionary and behavioral biologists [12]–[16], little is known about behaviors not directly associated with mate choice [12], [13], [15], [16; for research related to X. birchmanni shoaling preferences], [see ref. 14]. Typical of poeciliids, *X. birchmanni* adults do not provide post-partum parental care. Thus, neonates are left to fend for themselves. In their native streams, *X. birchmanni* fry likely experience predation from multiple sources including several piscivorous fishes [17], [18]. Here we use the sympatric native cichlid *Herichthys carpintes* to investigate the use of the chemical and visual cues of likely predators by *X. birchmanni* fry. If fry use chemical cues in predator detection, we predict that when fry encounter predator chemical cues they will respond with shoal tightening – tighter shoaling in fish, flocking in birds, and herding in mammals, are adaptive responses to the presence of potential predators [reviewed in refs. 2]–[4] – and with defensive behaviors such as seeking out corners and walls of the test tanks. Also, if fry use chemical cues in interactions with adult conspecifics, and if the presence of adult conspecifics signals the presence of food and/or an absence of predators, then we predict that fry will be most attracted to adult conspecifics when they can see and smell them.

## Methods

On 22 and 24 April 2006, we used seines and baited minnow traps to capture *X. birchmanni* adults and fry (∼4–10 mm), and three mature *H. carpintes,* on the Río Garces, Hidalgo, Mexico (20°57′22 N, 098°16′48 W). All individuals were transported immediately to the Centro de Investigación Cientifica de las Huastecas “Aguazarca” (CICHAZ) in Calnali, Hidalgo, Mexico, where preference/avoidance trials were conducted within three days of capture. Based on growth curves of closely related *Xiphophorus* [19], the fry used in the experiment were less than 7 days old.

**Figure 1:**
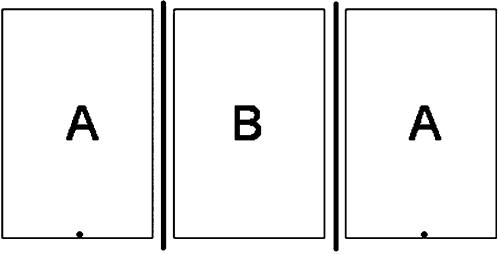
Schematic of the Experimental Setup Two pairs of fry – one in each compartment ‘A’ – were placed on either side of the visual stimulus tank (B). During trials with visual stimuli, the opaque dividers (thick black lines) were removed, and fry could see either a single large cichlid – in PV and PCV trials – or two adult conspecifics – in CV and CCV trials. In trials involving chemical stimuli, stimulus water was dripped into one end of the fry tanks (represented by the black point).

Prior to placing fry in ‘test tanks’ (28 cm×17.5 cm×17 cm), each tank was filled to a depth of approximately ∼2.30 cm with tap water treated with Prime (Seachem Laboratories Inc., Covington, Georgia, USA), and placed on a piece of graph paper, allowing us to quantify fry location at different periods during each trial. The test tanks were placed on opposite sides of the visual stimulus tank (same dimensions as test tanks) (Figure 1), and then one pair of fry was placed in each test tank. Between trials, during acclimatization periods, and during chemical stimulus trials not involving a visual stimulus presentation, removable opaque dividers visually separated test tanks from the visual stimulus tank (Figure 1). The chemical stimulus tank – identical dimensions as test tanks – was filled with chemical stimulus water, and was placed approximately 0.5 m above the test tanks and the visual stimulus tank (Figure 1). To make predator chemical stimulus water, the three cichlids were housed for more than 24 hours in a standard 40 l aquarium; water from this tank was then used in all predator chemical stimulus trials. To make conspecific chemical stimulus water, *X. birchmanni* adults were housed in tubs filled with ∼40 l of treated tap water; approximately 50 adults were housed in each tub for at least 24 hours before the water was used for conspecific chemical stimulus trials. Adults were not fed during the housing period. All trials were recorded using a Sony VX2000 digital video camera, mounted approximately 0.75 m above the test and visual stimuli tanks.

We separated our 36 fry into 16 pairs, haphazardly chosen – every two fry caught with a sweep of the dip net became a pair for the experiment. Each pair of fry was presented with a full suite of stimulus presentations (see below), and was used only once; following the final stimulus presentation, each fry pair was into an awaiting post treatment 40 l tank. Sets of fry pairs – four fry in total (see Figure 1) – were presented with a series of six stimulus presentations: conspecific chemical (CC), conspecific visual (CV), conspecific chemical and visual (CCV), predator chemical (PC), predator visual (PV), and predator chemical and visual (PCV); for each set of fry pairs, the order of stimulus presentations was randomized to control for possible order effects. In CV and CCV presentations, one adult male and one adult female were placed in the visual stimulus tank. In PV and PCV presentations, one large (∼5.0 cm) cichlid was placed in the visual stimulus tank, between the fry test tanks on either side (Figure 1). After placing one pair of fry in each test tank, trials began with a five minute acclimatization period, followed by a five minute stimulus presentation. After visual stimulus presentations, the opaque dividers separating the test tanks from the visual stimulus tank were replaced. During chemical stimulus presentations, chemical stimulus water was dripped into each test tank at constant rate; following chemical stimulus presentations, fry were removed from test tanks, the tanks were rinsed and then refilled with treated tap water, and fry pairs were returned to test tanks. All stimulus presentations were preceded by a five minute acclimatization period.

Video recording began at the start of each acclimatization period, and continued through the proceeding five-minute stimulus period; twice during each trial we recorded still images to determine fry locations. These images of fry locations were taken 30 seconds prior to stimulus presentations (i.e. during the acclimatization period), and 30 seconds after stimulus presentations began. At each time period, we determined fry proximity to (i) each other, (ii) the stimulus (for chemical stimulus presentations, we calculated the distance of each fry from the chemical water input tube; for CV presentations, we used the distance of each fry from the nearest adult), (iii) the nearest corner of the test tank, and (iv) the nearest wall of the test tank. For analyses, we pooled measurement data across fry pairs so that we could compare mean distances of fry from each other, from the stimulus, and from tank walls and corners, among treatments. ANOVA were used to evaluate treatment effects on fry proximity to tank walls, tank corners, and each other. If an ANOVA revealed significant treatment effects, Fisher LSD tests were used to evaluate differences between specific means [20]. Because the visual stimulus tank was not on graph paper, we could not calculate precise distances of fry from the visual stimulus animal, either a cichlid or nearest adult conspecific, thus we used discrete measures of distance: tank widths. For each visual stimulus trial, we calculated the number of half-tank widths between the fry and the visual stimulus animal; in the cases of CV and CCV trials, we determined the distance from the fry to the nearest adult. We pooled data among pairs of fry, and for each trial calculated the mean tank-width distances from visual stimuli. An ANOVA was used to evaluate treatment effects, and Fisher LSD tests were then used to evaluate differences between specific means [20]. We did not replicate the predator trials for two primary reasons. First, housing multiple predators is logistically problematic. Second, It has been argued that prey animals' responses to the presence of predators is under strong selection to be a highly generalized and stereotypical response – though in some cases requiring experiential learning to become stereotypical. This argument is supported by the common usage of single individuals as predator stimuli in previous studies of predator avoidance behavior in fishes [7], [9], [24]. For these reasons, we did not find it necessary to conduct replicated trials using multiple cichlids as predator stimuli.

## Results

**Figure 2:**
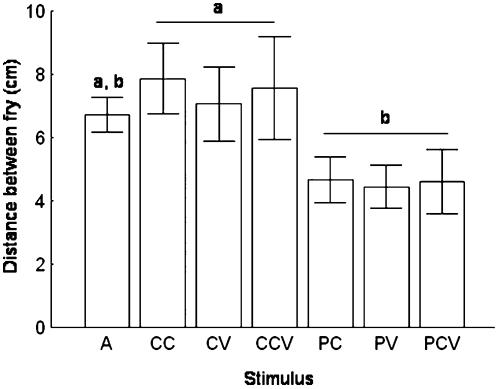
Mean Distance Between Fry During Trials This figure shows that fry shoal more tightly when presented with predator chemical (PC), predator visual (PV), and simultaneous predator chemical and visual (PCV) stimuli, than when presented with conspecific chemical (CC), conspecific visual (CV), and conspecific chemical and visual (CCV) stimuli. The mean distance between fry during the acclimatization period (A) was intermediate between the mean inter-fry distances during conspecific and predator stimuli trials. Bars and whiskers represent the mean±SE. Means with different letters above them are significantly different (*p*<0.05).

We tested the hypotheses that *X. birchmanni* fry use visual and chemical cues in detection of predators and conspecifics. We found that stimulus presentation did not affect fry proximity to the nearest wall (*F*_1,6_ = 1.59, *p* = 0.16) or corner (*F*_1,6_ = 1.43, *p* = 0.21) of the test tank, but did have a significant effect on fry shoaling (*F*_1,6_ = 2.24, *p* = 0.04). Fry responded to predator visual and chemical stimuli with shoal tightening (Figure 2).

**Figure 3:**
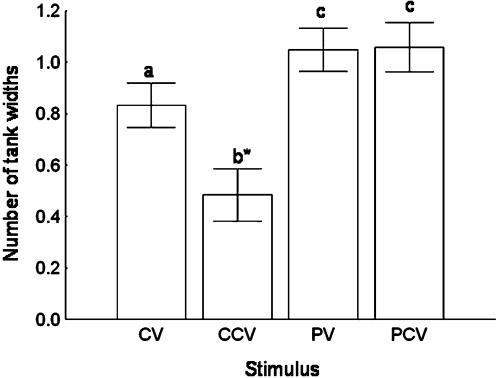
Comparisons of Mean Distances from Visual Stimuli This figure shows that fry (1) stayed further away from predators than they did from adult conspecifics, and (2) were most attracted to adult conspecifics when they could see and smell them (in conspecific chemical visual trials (CCV)). Bars and whiskers represent the mean±SE. Means with different letters above them are significantly different (*p*<0.05; asterisk: *p*<0.01).

ANOVA revealed that the type of stimulus affected fry distances from visual stimuli (*F*_1,3_ = 8.16, *p*<0.0001). Comparisons of mean distances from visual stimuli revealed that fry avoided predators, but were attracted to adult conspecifcs, especially when they could see and smell them (Figure 3).

## Discussion

Individuals in prey species are expected to attend closely to predator cues, and respond to these cues with predator avoidance tactics. This hypothesis has received much support among studies of adult fish [7], [8], [21; reviewed in refs. 22], [Bibr pone.0000118-Smith1] [23], and in a handful of studies investigating the use of predator chemical cues by juvenile fish. In the European perch (*Perca fluviatilis*), for instance, young-of-the-year reduce food intake when in the presence of odors of northern pike (*Esox lucius*) – a common perch predator [9]. And, in what reflects the long coevolutionary history between trout fry and their sympatric predators, young arctic char (*Salvelinus alpinus* L.) are able to discriminate among multiple predators based solely on chemical information, and modify their behavior adaptively based on olfactory information [24]. Here we show that week-old *Xiphophorus birchmanni* fry use a multimodal combination of chemical and visual cues in both predator detection and in interactions with adult conspecifics.

In this experiment, we found that fry responded to predator visual stimuli, chemical stimuli, and a combination of visual and chemical stimuli with tighter shoaling behavior than when presented with stimuli from adult conspecifics (Figure 2). Shoaling in response to predator proximity may benefit potential prey in several ways. First, shoaling may reduce the hunting efficiency of visual orienting predators that are confused in the presence of multiple targets [25], [26]. Second, by shoaling, the likelihood that a particular individual is attacked decreases with increasing shoal size. This adaptive explanation – i.e. the ‘selfish herd’ model – has been used to explain the widespread occurrence of grouping in many animals as a predator avoidance strategy [reviewed in refs. 25], [26]. Third, in species where experience is important in predator avoidance – such as in two-spotted gobies [7] – an inexperienced individual may benefit by associating with more experienced individuals who have acquired important predator avoidance behaviors.

We also found that *X. birchmanni* fry attend to the chemical cues of conspecific adults, and are particularly attracted to them when presented with simultaneous chemical and visual stimuli (Figure 3). One explanation for these results is that fry are attracted to the odors of conspecifics because they contain cues indicating the presence of food. More specifically, we suggest that the odors may contain information about the success of the particular adults at acquiring food. *Xiphophorus birchmanni* can smell the nutritional state of conspecifics: a recent study of mate choice in *X. birchmanni* showed that females prefer the odors of well-fed males to the odors of food-deprived males [15]. We suggest that *X. birchmanni* fry may attend to the same cues indicating nutritional state, though use the information in decisions related to resource acquisition, not mate choice.

Our findings that *Xiphophorus birchmanni* fry use chemical cues in predator detection and in interactions with conspecifics may have important implications for conservation of this species. Recent research on mate choice in *X. birchmanni* has shown that (i) females use chemical cues in mate choice [13], and (ii) anthropogenic alteration of the chemical environment disrupts the chemical communication system used in mate choice [15]. More specifically, Fisher et al. [15] showed that females lose their species-specific mate preferences in the presence of anthropogenic chemical inputs, such as sewage and washing detergents. Because fry likely use the same chemosensory pathways to detect predators, and conspecifics, alteration of the chemical environment may similarly disrupt predator detection and resource acquisition.

Chemical information is a ubiquitous feature in most environments [27]. It is therefore not surprising that animals have evolved diverse sensory systems for collecting, detecting, and processing chemical stimuli [27]. Here we show that very young animals have sophisticated mechanisms for discriminating among specific chemical cues, and that they are able to process information from multiple sensory modalities resulting in adaptive behavioral responses.
